# Can ambient odors influence the recognition of emotional words? A behavioral and event-related potentials study

**DOI:** 10.1007/s11571-021-09733-7

**Published:** 2021-11-02

**Authors:** Danyang Li, Xiaochun Wang

**Affiliations:** School of Psychology, Shanghai University of Sport, 650 Qing Yuan Huan Road, Yangpu District, Shanghai, 200438 China

**Keywords:** Olfaction, Odor context, Emotional word, ERP, Pleasant odor, Unpleasant odor

## Abstract

Odor context can affect the recognition of facial expressions. However, there is no evidence to date that odor can regulate the processing of emotional words conveyed by visual words. An emotional word recognition task was combined with event-related potential technology. Briefly, 49 adults were randomly divided into three odor contexts (pleasant odor, unpleasant odor, and no odor) to judge the valence of emotional words (positive, negative, and neutral). Both behavioral and Electroencephalography (EEG) data were collected. Both the pleasant odor and unpleasant odor contexts shortened the response time of the subjects to emotional words. In addition, negative words induced greater amplitudes of early posterior negativity (EPN) and late positive potential (LPP) than the positive and neutral words. However, the neutral words induced a larger N400 amplitude than the positive and negative words. More importantly, the processing of emotional words was found to be modulated by external odor contexts. For example, during the earlier (P2) processing stages, pleasant and unpleasant odor contexts induced greater P2 amplitudes than the no odor context. In the unpleasant odor context, negative words with the same odor valence induced greater P2 amplitudes than the positive words. During the later (N400) stages, various regions of the brain regions exhibited different results. For example, in the left and right frontal areas of the brain, exposure to positive words in a pleasant odor context resulted in a smaller N400 amplitude than exposure to neutral words in the same context. Meanwhile, in the left and right central regions, emotional words with the same valence as pleasant or unpleasant odor contexts elicited the minimum N400 amplitude. Individuals are very sensitive to emotional information. With deeper processing, different cognitive processes are reflected and they can be modulated by external odors. In the early and late stages of word processing, both pleasant and unpleasant odor contexts exhibited an undifferentiated dominance effect and could specifically modulate affectively congruent words.

## Introduction

Generally, people feel relaxed and happy when they smell the fragrance of flowers, and they feel disgusted when they smell moldy food. Thus, it is apparent that exposure to odor affects mood. It has been hypothesized that biological adaptations have resulted in prioritization of pleasurable stimuli, as well as emotionally threatening stimuli. Correspondingly, odors associated with unique smell could potentially influence people's behavior. Induction of higher cognitive processing due to the direct influence of odors could further affect people's emotions (Collet et al. 1997). It has been demonstrated that brain structures such as the amygdala, hippocampus, orbitofrontal cortex (OFC), and insula (Neville and Haberly 2004) are involved in olfactory processing. They are also the main structures for emotional processing (Dolan 2002). The connection between these two processing mechanisms makes them inseparable, and the connection between olfaction and emotion has gradually been attracting the attention of the scientific community.

The promotion or conflict of multi-sensory information interaction had been concerned in several studies, such as vision with hearing (Delogu et al. 2019). Because of the unique connection between olfaction and emotion, whether olfaction would be associated with other senses for emotional connection had also been investigated in a variety of ways, among which the emotional interaction between olfaction and vision was closer. Many studies had shown that when people smelt different odors, the valence scores or recognition speed of facial or bodily expression showed different performance. For example, in a study conducted by Cook et al. (2017), odors were found to enhance assessed valence in an interactive manner (e.g., disgusting faces are perceived as more negative in an unpleasant odor environment) (Cook et al. 2017). Previously, Cook et al. (2015) demonstrated that odor valence affects evaluations of the valence of neutral faces (Cook et al. 2015). Food odors can also affect the recognition of facial expressions. For example, unpleasant food odors prompt people to recognize facial expressions more quickly, especially fearful expressions (Li et al. 2020). Li and Wang also found that unpleasant odor context promoted the recognition of bodily expressions (Li and Wang 2021). Preliminary neurological evidence further suggests that emotional odors modulate responses of the visual cortex to emotional faces (Forscher and Li 2012; Seubert et al. 2010).

All of the above studies prove that cross-sensory emotional communication exists between smell and vision. Furthermore, all of the visual materials used in these experiment were pictures. Words are a common way to emotionally communicate in daily life. For example, we often need to judge and understand the emotional state and thoughts of others by their written words. But to date, there had been few investigations about how odors affected the recognition/understanding of emotional words. There are many differences between words and pictures. Pictures may be more visually prominent and/or processed faster, while words may require more supporting resources (Wood et al. 2016). Words can also represent generic names of types, while pictures show specific instances of types (Lai et al. 2012). Importantly, words are thought to be less perceptually complex than pictures, which may lead to different emotional meanings in a person's biological readiness (Kissler et al. 2006). Therefore, the pattern of smell which affects the processing of emotional words may differ from the processing used for emotional pictures, and this difference needs to be further explored.

Not only that, the neural mechanisms that odors regulate the processing of emotional words are not yet understood. At present, researchers have demonstrated an influence of odors on visual processing of facial and bodily expressions, as well as on its time-course (Li et al. 2020; Li and Wang 2021). However, few studies have used emotional words as visual objects and combined them with smell. For example, in the early component of the ERP components, investigator found that, the amplitude of P2 induced by expressions of disgust and happiness was found to be lower than that for neutral expressions under unpleasant odor conditions (Leleu et al. 2015). In the later component of Event-related potential (ERP) components, Cook et al. (2017) found the consistency effect in N400 (Cook et al. 2017), that is, the amplitude of happy faces was larger than that of disgusted faces in the presence of an unpleasant odor, while disgusted faces had a greater amplitude than happy faces in the presence of a pleasant odor. However, odor was found to elicit an unstable response at the late positive potential (LPP). In a study, when individuals were exposed to human sweat collected during anxiety, participants' LPP response was increased in response to neutral and fuzzy facial expressions (Rubin et al. 2012). In contrast, when individuals were exposed to scents associated with threat and danger, they evaluated faces more often as negative, yet no significant LPP effect was observed (Kastner et al. 2016). Li’s study (2021) showed that VPP amplitudes induced by bodily expressions were greater in an unpleasant odor context than in a pleasant odor context. And when exposed to an unpleasant contextual odor, N2 and LPP amplitudes related to fearful bodily expressions were smaller than when exposed to other odor contexts (Li and Wang 2021).

Event-related potential (ERP) components related to the processing of emotional words include early p1-N1 components, middle P2 and early posterior negativity (EPN) components, and later N400 and LPP components. Initially, it was reported that emotional words had a larger amplitude than neutral words in the early components within 100 ms (Scott et al. 2009). Other researchers have found that the P2 amplitude that was induced by emotional words was significantly greater than that induced by neutral words (Herbert et al. 2006; Kanske and Kotz 2007). EPN, which is mainly distributed within the occipitotemporal region, is often mentioned in research of emotional words. It has been observed that emotional words trigger greater EPN amplitudes than neutral words (Kissler and Herbert 2013; Zhang et al. 2014), while other studies have reported differences between positive and negative words (Bayer and Schacht 2014; Espuny et al. 2018; Schacht and Sommer 2009a). At the late stage of word processing, a semantic analysis of emotional words is usually faster than that of neutral words, and at the same time, emotional words induce N400 with a smaller amplitude (Kanske and Kotz 2007). LPP generally begins 300 ms after presentation of a stimulus and it lasts for an extended period of time, thereby reflecting that an individual is undergoing further cognitive processing, as well as continuous attention, stimulus assessment, and memory (Hajcak et al. 2010). Relevant ERP studies have found that emotional words have a larger LPP component than neutral words (Fischler and Bradley 2006; Herbert et al. 2006). Therefore, in order to explore whether odor contexts can influence the visual recognition of emotional words and their mechanism, it is necessary to investigate changes in ERP components that are closely related to emotional words and to further expand the theoretical path.

Current research lacks evidence regarding behavioral and neural mechanisms of the effect of smell on the visual processing of emotional words. These mechanisms are not only related to the field of research of information communication between olfactory and visual cross-sensory channels, but can also further reveal people's processing preferences when they interweave different emotional information. At the same time, it is inevitable for people to deal with the emotions expressed by visual words more efficiently in order to better understand the original intention of another party. Therefore, the aim of this study was to explore whether different odor contexts can help people identify emotions, thereby demonstrating whether odor environments can facilitate social interactions.

## Methods

### Participants

We used G* Power 3.1.9.4 to estimate the sample size required. According to the study of Cohen (1988) and Zhang (2020) (Cohen 1988; Zhang et al. 2020), we set a medium effect size F (the value is 0.25), given the α value (0.05), power value (0.80), and a total sample size of 45 was needed, i.e., at least 15 people was assigned to each odor group. A total of 49 healthy adult subjects (24 males, 25 females) participated in this study. They ranged in age from 18 to 30 years, with an average age of 22.24 years (standard deviation (SD) = 2.64). All of the participants were healthy, right-handed, and had normal or corrected-to-normal vision. None of the subjects had a history of brain injury or neurosis, nor had they reported acute nasal infections or allergies that affected their sense of smell. All of the subjects signed informed consent and privacy policy forms. This study was conducted in accordance with the Declaration of Helsinki and was approved by the Ethics Committee of Shanghai Sport University (102772019RT004). Upon completion of the experiments, the subjects received remuneration.

### Materials

#### Word stimuli

Various words were used from the Chinese Affective Words System (CAWS) (Yi-niu. et al. 2008): 34 positive words (valence: > 6.65 on the 9-point scale), 34 negative words (valence: < 3.29), and 34 neutral words (valence: 4.61–5.63) were selected. Each category of emotional words included 17 nouns and 17 adjectives, and all of them were two-character words. Before the formal experiment was started, 28 subjects (13 males, 15 females; mean age = 23.82 ± 1.58 y) who did not participate in the Electroencephalography (EEG) experiments were recruited for a preliminary experiment. These subjects were asked to assess the valence and arousal of words from low to high on a 9-point self-assessment manikin (SAM) scale. The obtained behavioral data were analyzed by one-way analysis of variance (ANOVA) with SPSS 22.0. As shown in Table 1, the main effect of valence (F (2, 99) = 1016.040, *p* < 0.001) and of arousal (F (2, 99) = 36.594, *p* < 0.001) were significant. Post-hoc tests identified significant differences in the pleasure levels of the three types of words (ps < 0.01). In terms of arousal, there were significant differences between positive and negative words and neutral words (ps < 0.05), yet there was no significant difference between the positive and negative words (*p* > 0.05). For the six types of words (positive, negative, and neutral × adjectives and nouns), there were also no significant differences in word frequency, familiarity, and total number of strokes (ps > 0.05) (Table 1).

**Table 1:** Characteristics of the word stimuli used

Quality	Word type, *M* (SD)		
	Positive	Neutral	Negative
Valence (range: 1–9)	6.86 (0.34)	5.16 (0.34)	2.98 (0.39)
Arousal (range: 1–9)	5.17 (0.70)	4.04 (0.58)	5.41 (0.81)
Word frequency	31.12 (41.27)	34.53 (38.44)	54.18 (119.54)
Familiarity	5.81 (0.59)	5.49 (0.66)	5.59 (0.65)
Total Strokes	14.68 (4.83)	15.82 (5.50)	15.15 (3.58)

#### Odor stimuli

The pleasant and unpleasant odors used in our formal experiment were selected from nine familiar odors: lemon, chocolate, apple, garlic, alcohol, durian, rotten fish, vanilla, and vinegar. Briefly, we recruited 17 individuals who were not involved in the formal experiment to rate the nine odors according to a 7-point SAM scale of pleasantness, intensity, and arousal. A score of 1 indicated a very unpleasant/low/weak smell, while a score of 7 indicated a very pleasant/high/strong smell. Based on this feedback, essential oil smelling of lemon (a 96% mixture of *cis* and *trans* purchased from Sigma-Aldrich, MO, USA) received the highest pleasantness score, while an essential oil smelling of rotten fish (Givaudan Inc., Geneva, Switzerland) received the lowest pleasantness score. In previous studies, odors of lemon and rotten fish were also regarded as positive and negative odors (Chen and Dalton 2005; Damjanovic et al. 2018). These essential oils were diluted with mineral oil (50%, v/v) and 1, 2-propanediol (42%, v/v), respectively, for use in the formal experiment. Meanwhile, air was used as a neutral odor stimulus (Boesveldt et al. 2010). According to one-way ANOVA, the lemon odor (M = 5.94, SD = 0.66), rotten fish odor (M = 2.12, SD = 0.86) and air (M = 4.00, SD = 0.00) were found to significantly differ in pleasantness and in arousal (*p* = 0.84). The lemon scent was found to be more pleasant than air (*p* < 0.001), and air was more pleasant than the rotten fish odor (*p* < 0.001). The lemon and rotten fish odor also received a higher arousal rating than air (*p* < 0.001), yet there was no difference between the lemon and rotten fish odor (*p* > 0.05). The odor which received a moderate score for intensity was selected to ensure that the odor concentration was relatively constant during the experiment. Ambient odor was distributed as an aerosol with use of a TLDQ-806 basic air odor diffuser (TLDQ, Shenzhen, China). Previous studies have shown that exposure to odors diffused in an environment is an effective method of stimulus delivery (Gilbert et al. 1997; Herz 1997; Ludvigson and Rottman 1989).

### Experimental desigh

A 3 × 3 mixed design was used in our experiment, with odor context (pleasant, unpleasant and no odor) as the between-subjects factors and emotional words (happy, fearful and neutral) as the within-subjects factors. The accuracy and response time of emotional words as the behavioral index and the mean amplitude of P2, EPN, N400, and LPP as the neuropsychological index were set as dependent variables. There were 17 participants in the pleasant odor group (8 females and 9 males), 17 participants in the unpleasant odor group (9 females and 8 males), and 15 participants in the non-odor group (7 females and 8 males).

### Experimental procedure

All of the subjects were tested in the same laboratory setting. Briefly, the subjects were randomly divided into three groups and placed in pleasant odor, unpleasant odor, and air control environments. Prior to conducting the experiments, each subject had to answer questions to indicate whether they were aware of the odor. Subsequently, the odor environment was rated on its pleasantness, intensity, and arousal by using a 7-point scale (e.g., ranging from very unpleasant to very pleasant; very weak to very strong; and not pungent to very pungent, respectively). The purpose of this step was to verify that the odors used were perceptible, and to perceive the degree of stimulation. Images used in the formal experiments were displayed on a screen at a resolution of 1024 × 768 pixels. E-prime 2.0 software was used to control stimulus presentation.

**Fig. 1:**
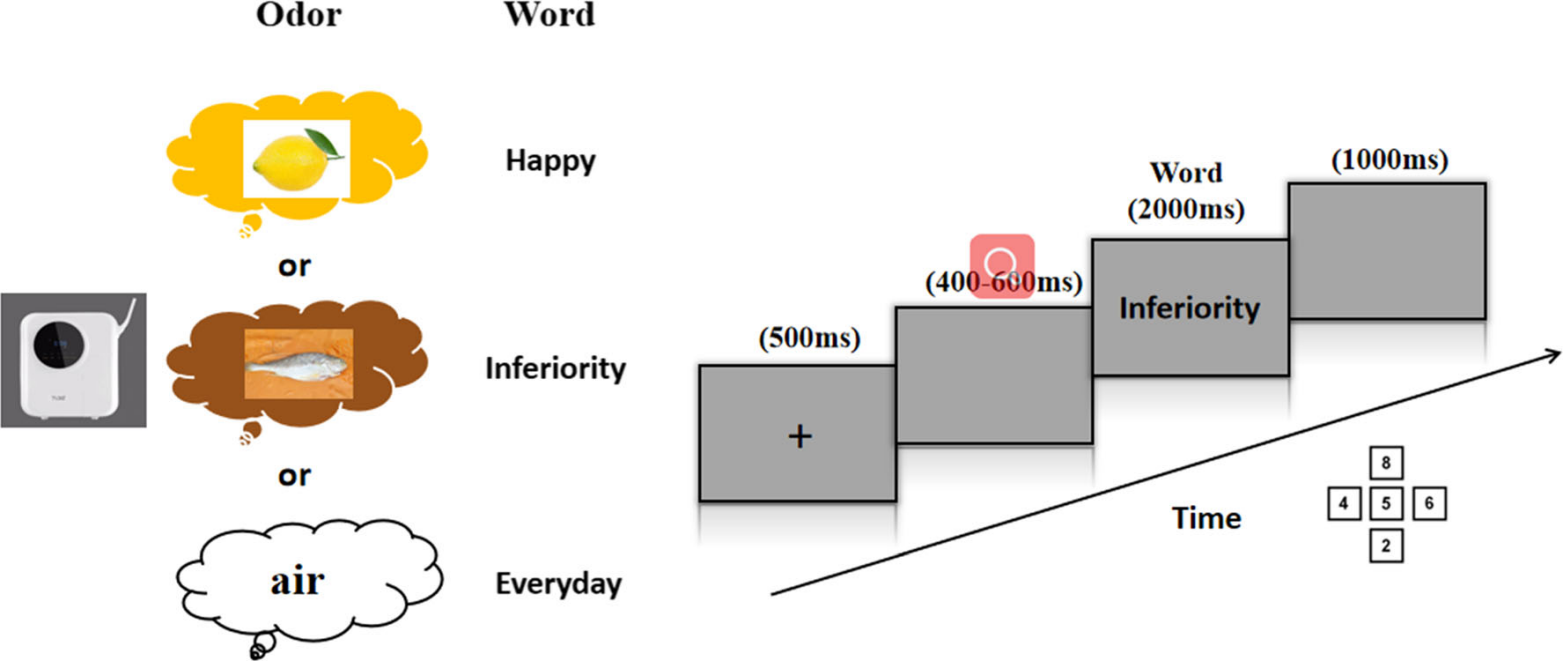
Left: Representative odors and words used in the experiments. Right: An example of the procedure

The experimental procedure is shown in Fig. 1. Briefly, the center of the screen was rendered with a fixed plus sign for 500 ms (ms). Then, a blank screen appears for 400–600 ms. When a word appeared at random after the blank screen, participants were asked to judge whether the emotional valence of the word was positive, neutral, or negative. Positive, neutral, and negative words corresponded to the "4", "5", and "6" keys on the keyboard and were pressed by the right index, middle, and ring fingers, respectively. The emotional valence of the words corresponding to the "4" and "6" keys was balanced between participants. If the software did not record any response over a period of 2000 ms, the computer automatically displayed a final blank screen for 1000 ms to indicate completion of a trial (Fig. 1).

Overall, our experiment was conducted in three blocks, with each block including 60 trials for a total of 180 trials. Within each block, each word was rendered only once and was repeated three times in the formal experiment. The subjects had a one-minute break between each block. Before the experiment, the subjects underwent 12 trial experiments to familiarize themselves with the experimental tasks. The total test time was approximately 15 min. At the end of the formal experiment, the subjects were asked to rate the pleasantness, intensity, and arousal of the odor environment on a 7-point scale. The purpose of this step was to test whether the sensitivity of the subjects to the odor environment was stable and whether the odor concentration was set appropriately by comparing the subjects’ ratings to the odor context before and after the experiment (Li et al. 2020; Li and Wang 2021).

### EEG recording

Brain Products Recorder software was used. A 64-conductor Ag/AgCl electrode cap extended by the international 10–20 system was used to collect EEG signals. Reference electrodes were placed on the bilateral mastoid connection, and TP9 and TP10 were selected as reference electrodes. A bandpass filter was set for 0.01–100 Hz, with a sampling frequency of 500 Hz/channel. To obtain pure brain waves, impedance of all the electrodes was set below 10kΩ.

### Data analysis

E-prime 2.0 was used to automatically record behavioral data such as accuracy and reaction time of emotional word recognition tasks under three odor contexts. Accuracy referred to the ratio of the number of times that subjects correctly judged the valence of emotional words and pressed the right key to the total number of keys pressed. The reaction time was the duration from the appearance of the emotional words to keys pressed correctly.

EEG data were analyzed by using Brain-Vision Analyzer 2.1 software (Brain Products GmbH, Gilching, Germany). Independent component analysis was used to correct eye movements and blinks (Jung et al., 2000). Low-pass filtering at 30 Hz and 24 dB/octave was applied. Amplitudes beyond ± 80 V were automatically rejected as artifacts. Data were extracted offline from a 200-ms baseline obtained prior to the onset of a stimulus and from a 800-ms interval after onset of a stimulus. Finally, the correct trials of each condition were overlapped and averaged. To improve the signal to noise ratio, the ERP waveform was overlaid no less than 30 times for each condition.

Based on relevant literature (Fields and Kuperberg 2012; Herbert et al. 2011; Pinheiro et al. 2016; Shestyuk and Deldin 2010; Watson et al. 2007) and the total average waveform of ERP data, we selected electrode points and a time window corresponding to each component. SPSS 22.0 was used to conduct repeated-measures ANOVA of the average amplitudes of the EEG data. Analysis of P2 (160–240 ms), N400 (300–450 ms), and LPP (450–650 ms) were performed for both the left and right hemispheres. The left and right hemispheres were divided into four regions: left frontal region (F1, F3, FC1, FC3), right frontal region (F2, F4, FC2, FC4), left central region (C1, C3, CP1, CP3), and right central region (C2, C4, CP2, CP4). Therefore, repeated-measures ANOVA for the EEG data included several factors: odor context (pleasant, unpleasant, neutral), emotional word (positive, negative, neutral), and brain region (left frontal area, right frontal area, left central area, right central area). EPN reflects rapid attention capture and the early processing for salient information, and it is an early negative wave distributed in the back of the brain (Kissler et al. 2007). Based on previous literature (Herbert et al. 2008, 2011; Kissler et al. 2007; Schacht and Sommer 2009b) and current EEG results, 200–300 ms was selected as the time window for EPN analysis and the posterior electrodes included: P7, PO7, P8, PO8, O1, and O2. For behavioral (accuracy and reaction time) and EPN data, 3 × 3 (odor context × emotional word) repeated-measures ANOVA was performed. In addition, 3 × 2 (odor context × order) repeated-measures multivariate analysis of variance was performed to analyze pleasantness, intensity, and arousal for the three odor groups before and after the experiment. The Green-house–Geisser epsilon correction was used to correct p-values and degrees of freedom. Post-hoc comparisons were performed with the Bonferroni correction (p < 0.05) whenever appropriate. Effect size was expressed as partial eta squared (η_p_^2^).

## Results

### Evaluation of perceptual stability of odor

**Fig. 2:**
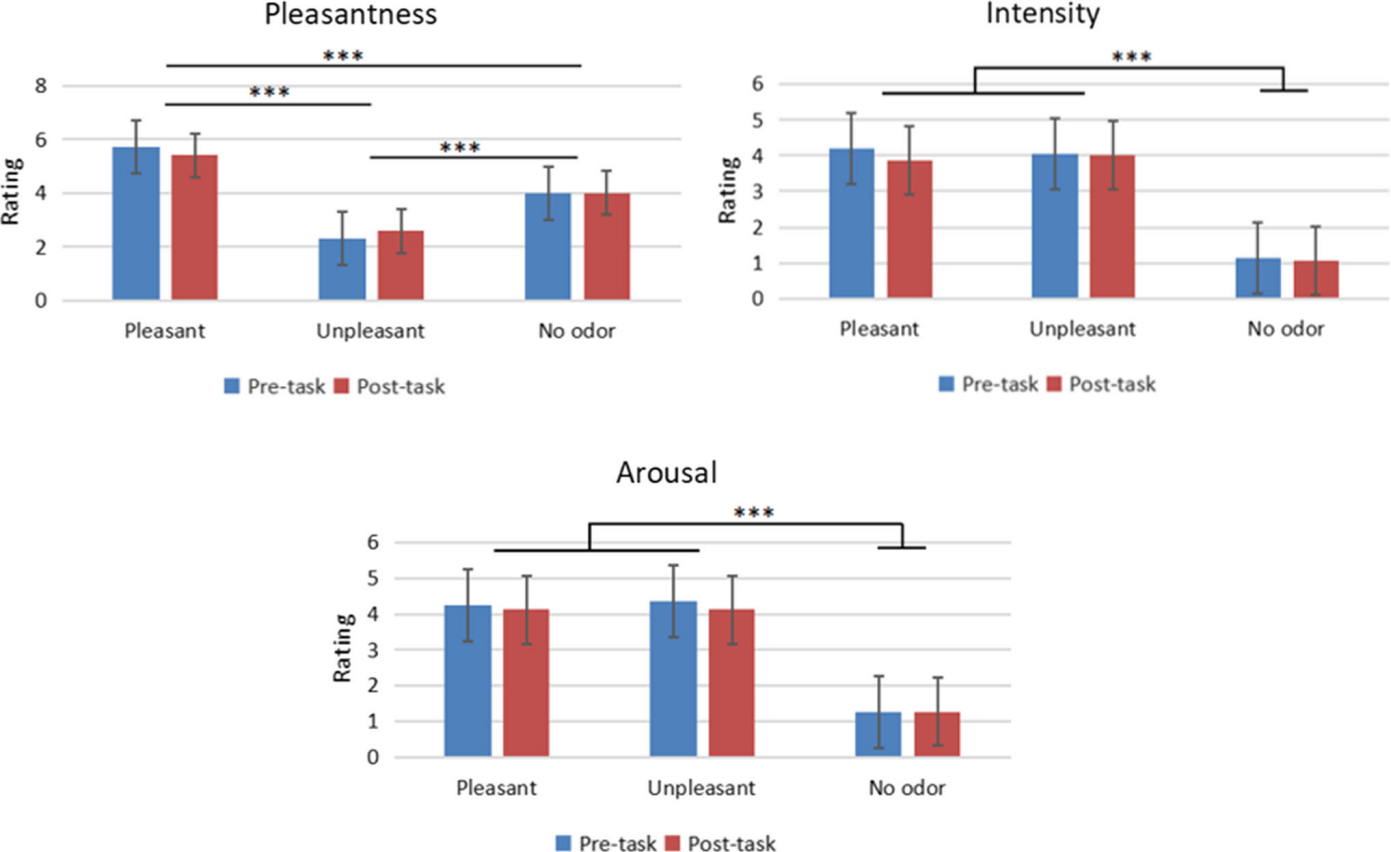
Mean pleasantness, arousal, and intensity ratings of the olfactory environment, as rated on a 7-point scale by subjects before and after each experiment. Error bars represent mean standard error. ***p < 0.001

There were no significant differences between pre- and post-task ratings of pleasantness, arousal, or odor intensity for the three odor contexts investigated (*p* > 0.05). The main effects were significant only in the odor environment (*p* < 0.001). Meanwhile, the pleasantness score was highest for the pleasant environment, followed by the no odor environment (*p* < 0.001) and the unpleasant odor environment (*p* < 0.001). Furthermore, the intensity and arousal scores of the pleasant and unpleasant odor environments were higher than those of the non-odor environment (each ps < 0.001) (Fig. 2).

### Behavioral results

**Fig. 3:**
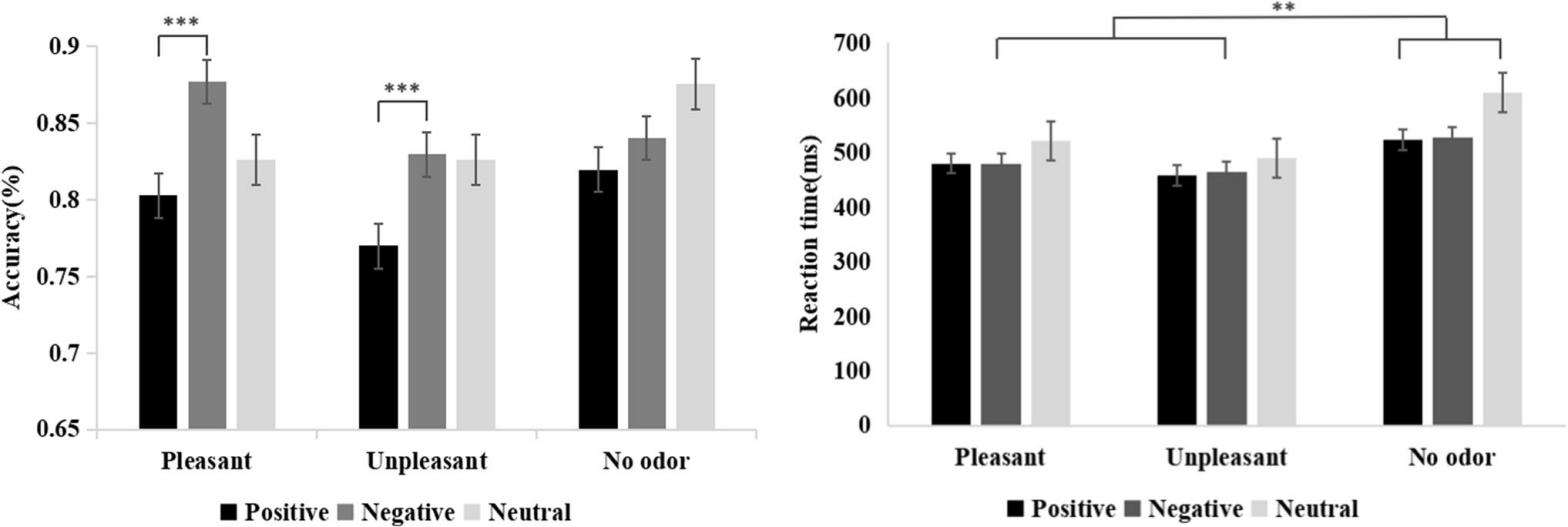
Average accuracy (left) and average reaction time (right) in each odor context × emotional word condition (error bars represent SE), **p < 0.01, ***p < 0.001

A 3 × 3 (odor context × emotional words) repeated-measures ANOVA revealed that response accuracy was significantly affected by emotional words [F (2, 92) = 4.087, *p* = 0.030, η_p_^2^ = 0.082]. Furthermore, the accuracy of negative words (0.849 ± 0.01) was higher than that of positive words (0.80 ± 0.01, *p* = 0.002). In contrast, the main effect of odor context was not significant [F (2, 46) = 2.315, *p* = 0.110, η_p_^2^ = 0.091]. Similarly, no significant interactions between emotional words and odor context were observed [F (4, 92) = 0.849, *p* = 0.474, η_p_^2^ = 0.036] (Fig. 3).

When a 3 × 3 (odor context × emotional words) repeated-measures ANOVA was performed for reaction time on emotional word recognition task, the main effect of emotional words was found to be significant [F (2, 92) = 19.231, *p* < 0.001, η_p_^2^ = 0.295]. Both positive (487.63 ± 10.05 ms, *p* < 0.001) and negative words (490.94 ± 10.51 ms, *p* < 0.001) had shorter reaction times than neutral words (540.89 ± 10.95 ms). In addition, the main effect of odor context was significant [F (2, 46) = 7.275, *p* = 0.002, η_p_^2^ = 0.240], and subjects reacted faster in both pleasant (494.19 ± 15.12 ms, *p* = 0.029) and unpleasant (471.57 ± 15.12 ms, *p* = 0.002) odor contexts than in the no-odor context (553.69 ± 16.09 ms). In contrast, interactions between emotional words and odor were not significant [F (4, 92) = 2.045, *p* = 0.095, η_p_^2^ = 0.082] (Fig. 3).

### ERP results

#### P2 (160–240 ms)

**Fig. 4:**
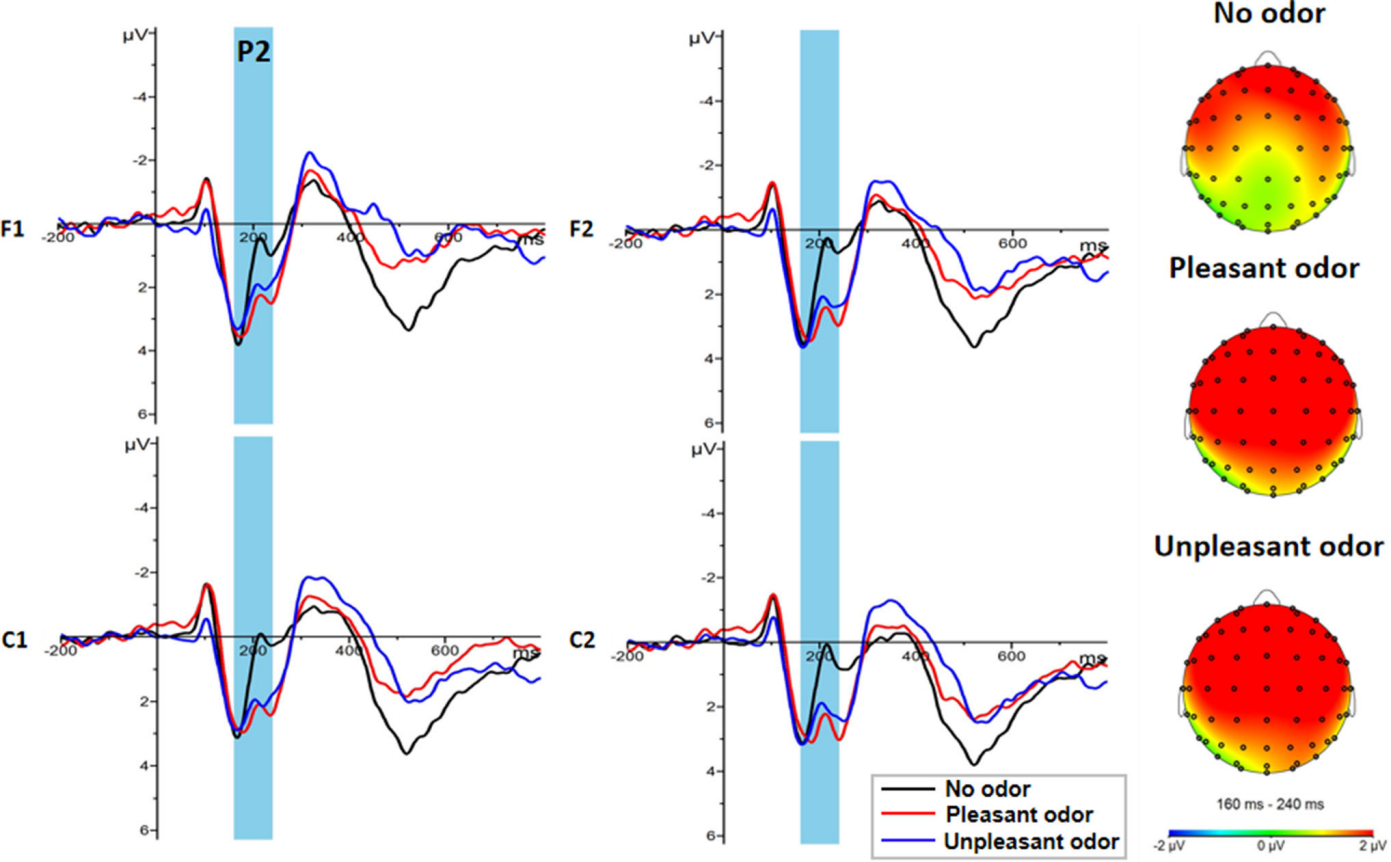
Grand average ERP waveforms and topography in odor context conditions. Left: Average ERP waveforms of P2 for no odor (black line), pleasant odor (red line) and unpleasant odor (blue line) recorded at electrodes F1, F2, C1 and C2. Right: Topography of P2 (160–240 ms) for odor condition. (Color figure online)

**Fig. 5:**
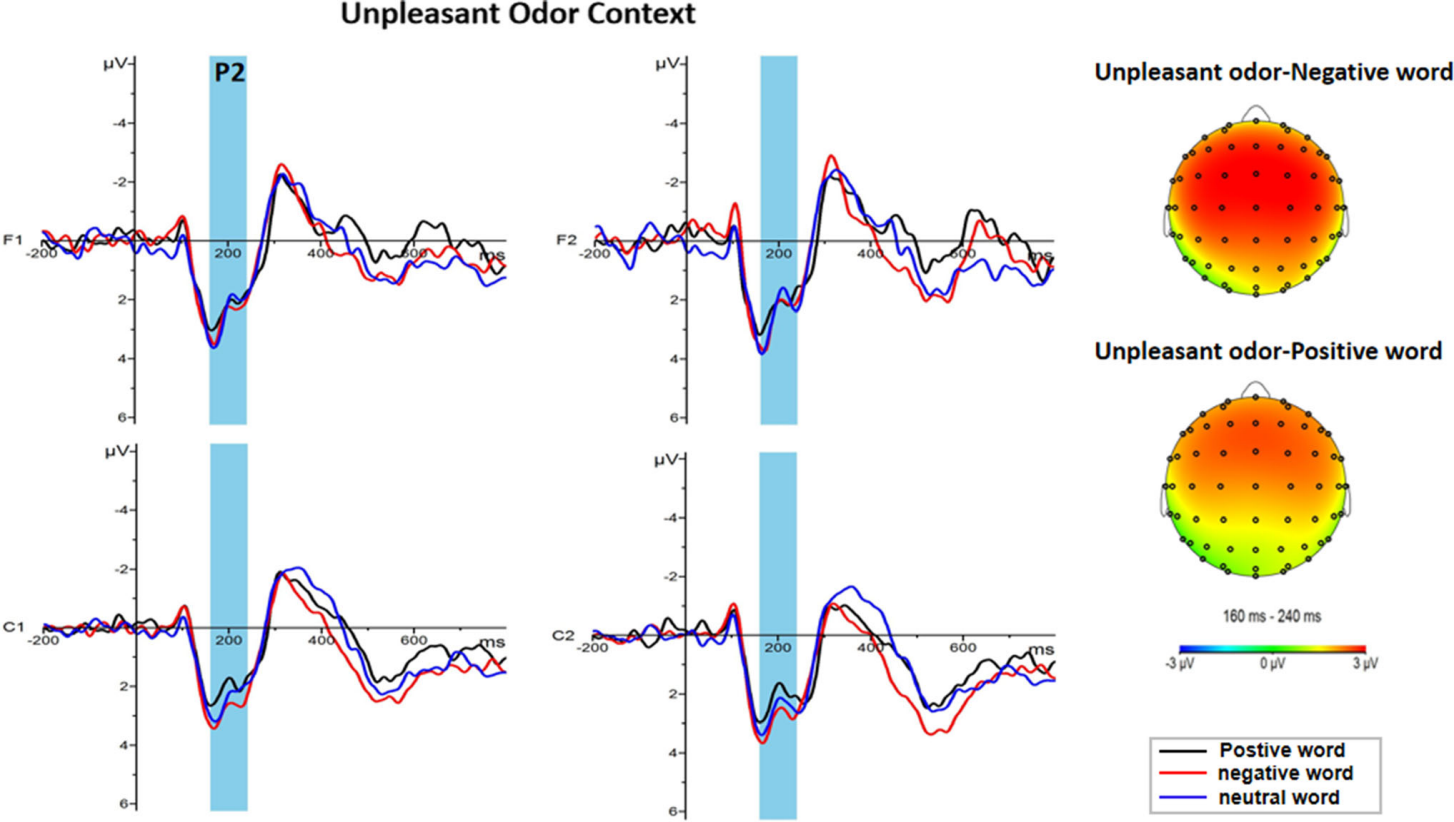
Grand average ERP waveforms and topography for three emotional words in unpleasant odor context. Left: Average ERP waveforms of P2 for positive (black line), negative (red line) and neutral word (blue line) in unpleasant odor context recorded at electrodes F1, F2, C1 and C2. Right: Topography of P2 (160–240 ms) for positive and negative words in unpleasant odor context. (Color figure online)

ANOVA results of the P2 component in both brain hemispheres showed that the main effects of odor context were significant [F (2, 46) = 4.632, *p* = 0.015, η_p_^2^ = 0.168]. Post-hoc tests revealed that emotional words observed in the context of a pleasant odor (2.80 ± 0.41μv, *p* = 0.037) or in the context of an unpleasant odor (2.86 ± 0.41μv, *p* = 0.028) both elicited significantly larger amplitudes than emotional words observed in a no odor context (1.25 ± 0.43 μv) (Fig. 4). Furthermore, the main effect of brain regions was significant [F (3, 138) = 10.135, *p* < 0.001, η_p_^2^ = 0.181], with the P2 amplitude greater in the left (2.55 ± 0.28 μv) frontal regions and right (2.66 ± 0.25 μv) frontal regions than in the left (1.99 ± 0.24 μv, *p* = 0.001) and right (2.02 ± 0.27 μv, *p* = 0.002) central region. A significant interaction between emotional words and odor context was also observed [F (4, 92) = 3.477, *p* = 0.011, η_p_^2^ = 0.131]. Simple effect analyses revealed that the P2 amplitude was larger for negative words (3.12 ± 0.44 μv) than for positive words (2.49 ± 0.39 μv) under the unpleasant odor condition (*p* = 0.033) (Fig. 5). In contrast, no significant main effect of other factors, or interactions between factors, were observed (all ps > 0.05).

#### EPN (200–300 ms)

**Fig. 6:**
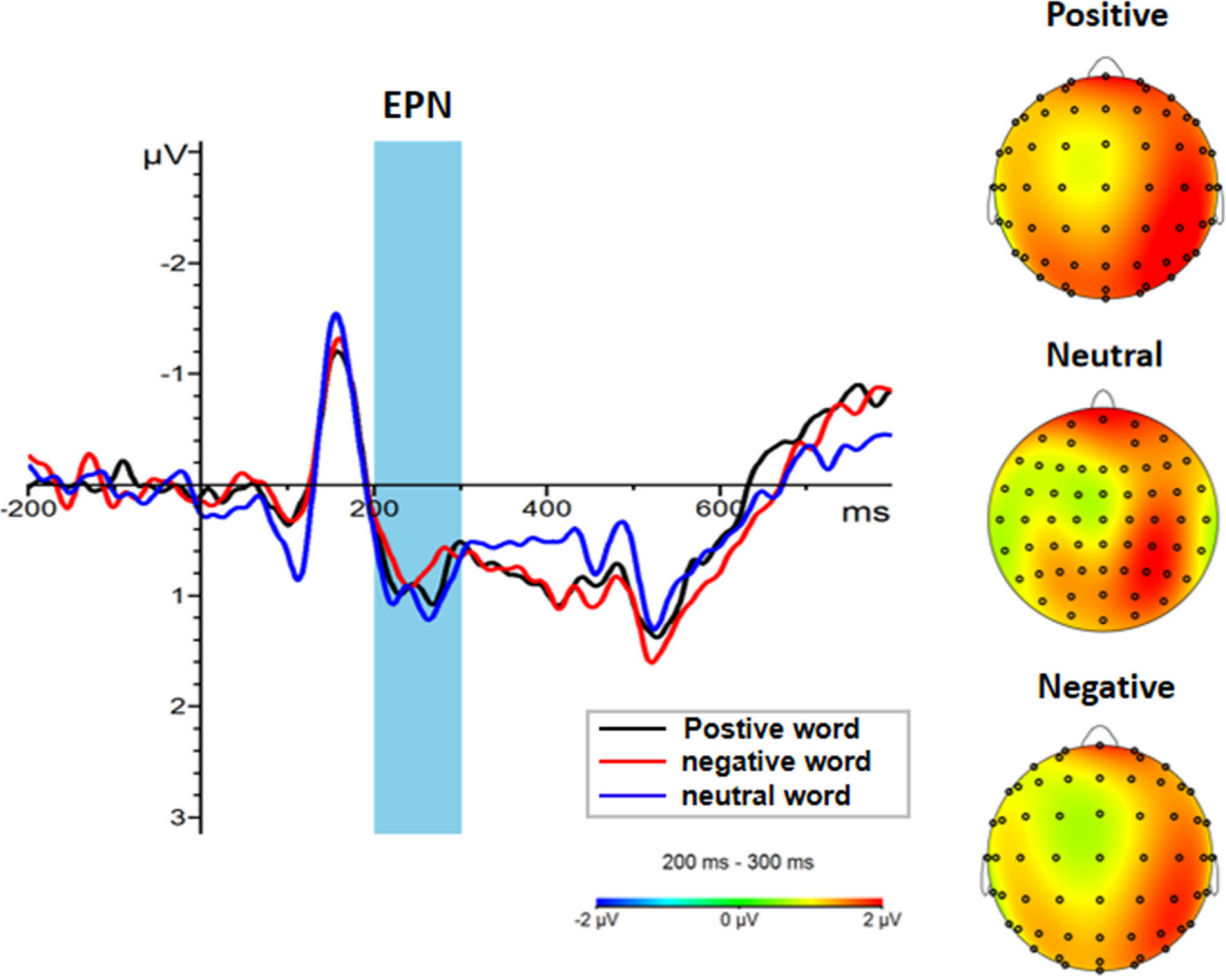
The amplitude and topography of EPN for emotional words condition. Left: Grand average ERP waveforms of EPN for positive (black line), negative (red line) and neutral words (blue line) recorded across electrodes P7, PO7, P8, PO8, O1, and O2. (B) Brain topography of EPN (200–300 ms) for emotional word condition. (Color figure online)

As shown in Fig. 3, a 3 × 3 repeated measures ANOVA for EPN component amplitude showed a significant main effect of emotional words [F(2, 92) = 5.854, *p* = 0.004, η_p_^2^ = 0.113]. Further analyses indicated that negative words (1.04 ± 0.20 μv) elicited a larger EPN than positive words (1.32 ± 0.22μv, *p* = 0.009) and neutral words (1.23 ± 0.20 μv, *p* = 0.009) (Fig. 6). In contrast, the main effect of contextual odor and the interaction between emotion words and contextual odor were not significant (all ps > 0.05).

#### N400 (300–450 ms)

**Fig. 7:**
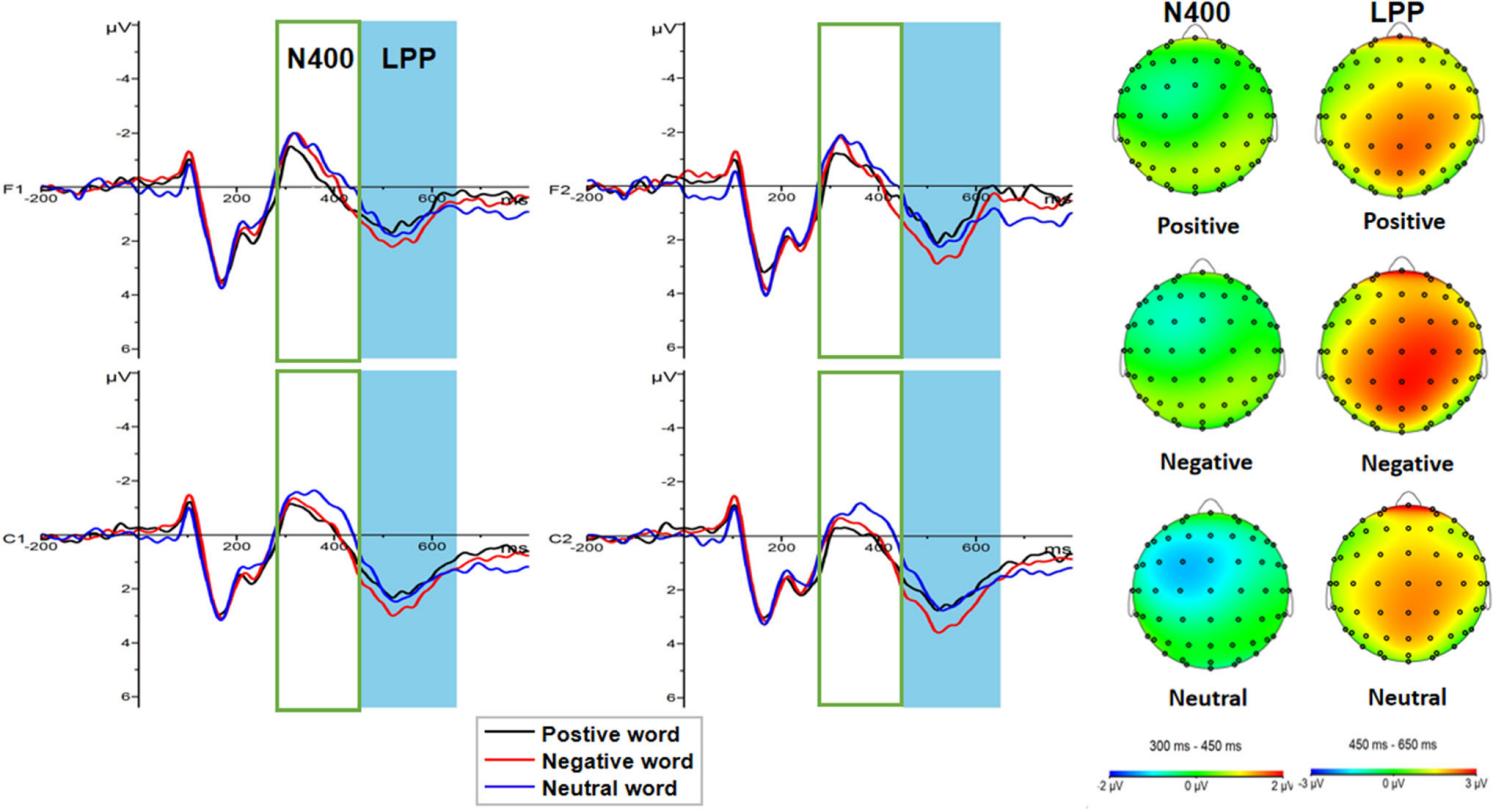
The amplitude and topography of N400 and LPP for emotional word condition. Left: Grand average ERP waveforms of N400 and LPP for positive (black line), negative (red line) and neutral word (blue line) recorded at electrodes F1, F2, C1 and C2. Right: Brain topography of N400 (300-450 ms) and LPP (450-650 ms) for emotional word condition. (Color figure online)

**Fig. 8:**
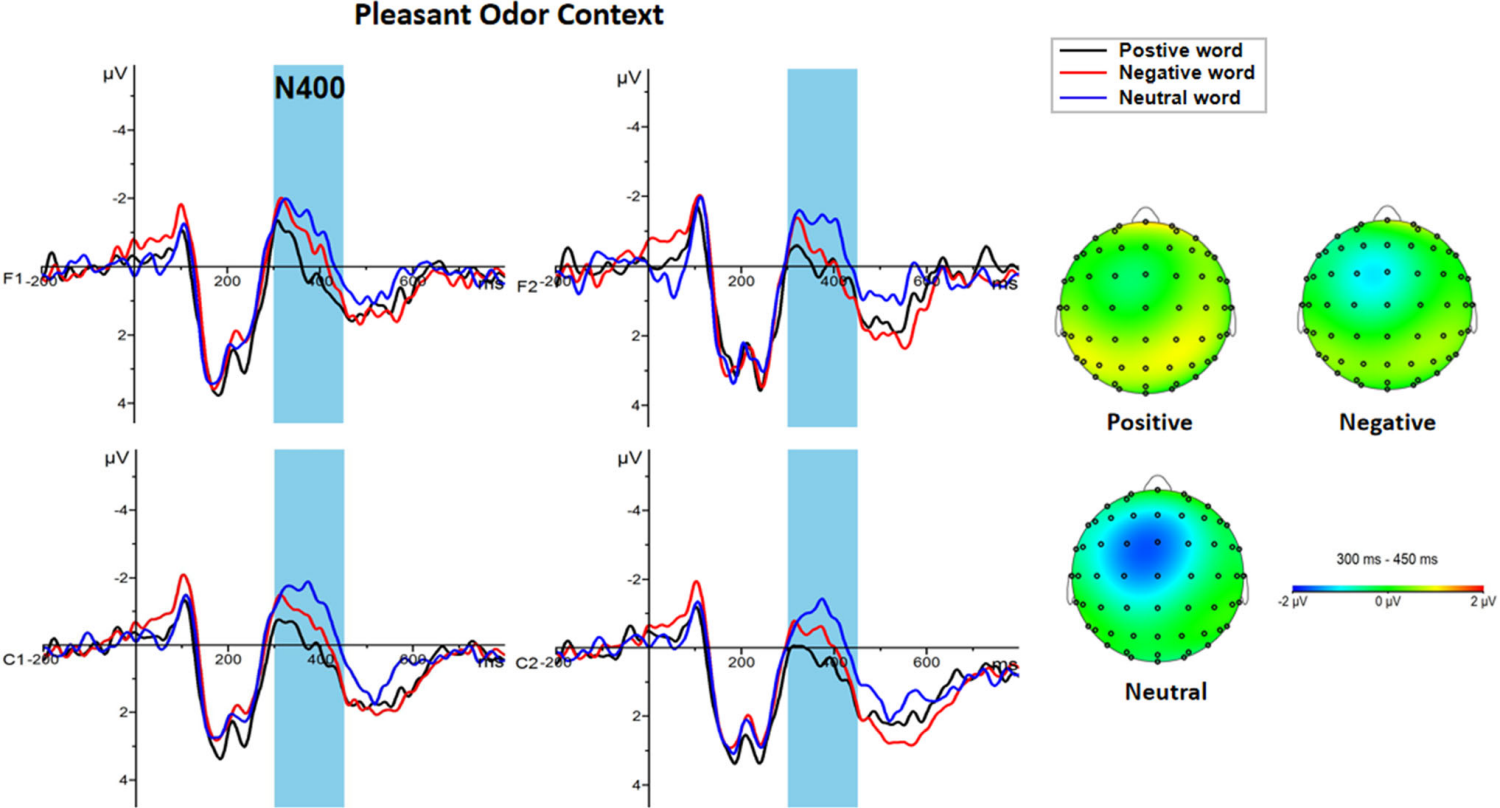
Grand average ERP waveforms and topography for three emotional words in pleasant odor context. Left: Average ERP waveforms of N400 for positive (black line), negative (red line) and neutral word (blue line) in pleasant odor context recorded at electrodes F1, F2, C1 and C2. Right: Topography of N400 (300–450 ms) for positive, negative and neutral words in pleasant odor context. (Color figure online)

**Fig. 9:**
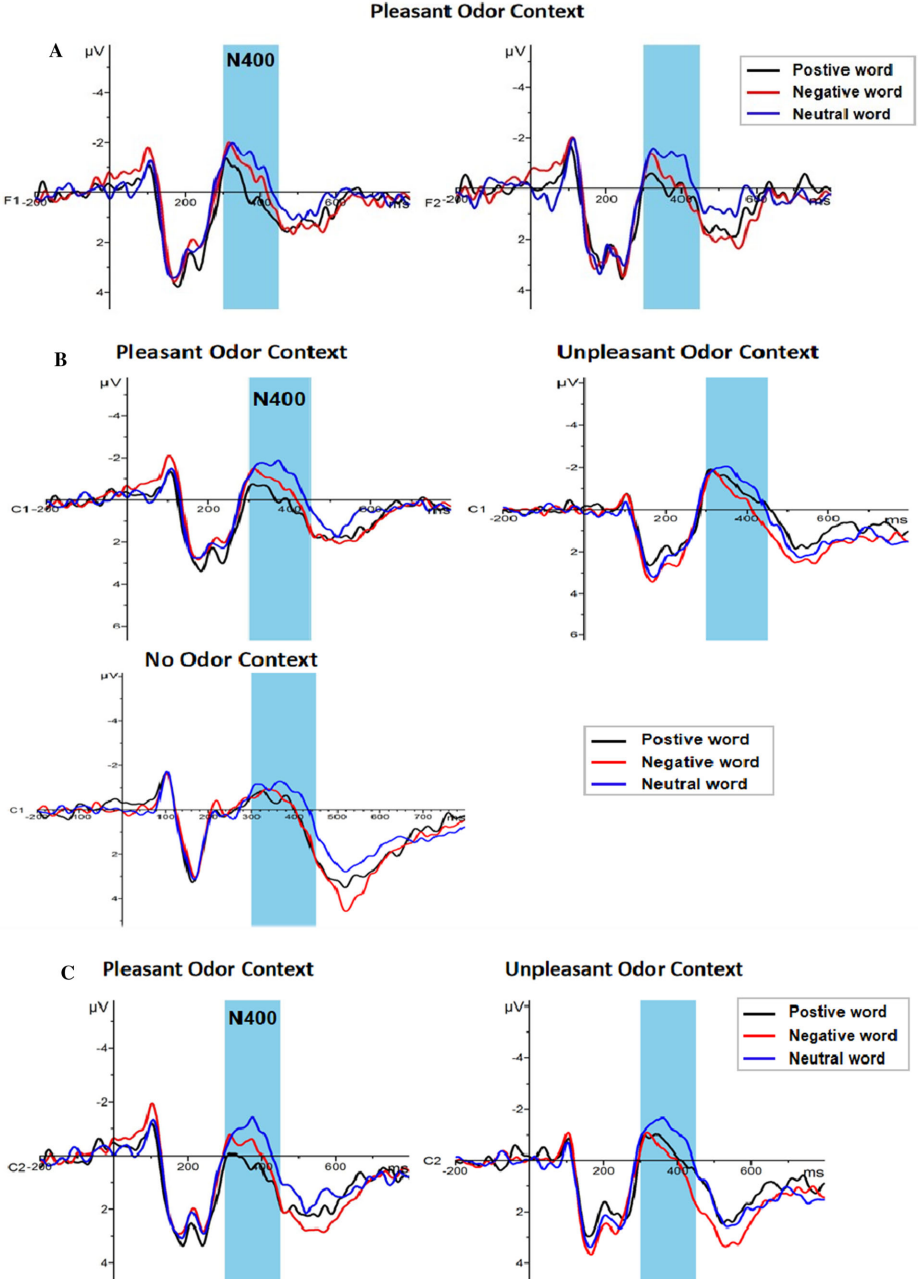
Interaction of emotional word, odor context and region for N400. **a** Average ERP waveforms of N400 for positive (black line), negative (red line) and neutral word (blue line) in pleasant odor context recorded at electrode F1 of left frontal region and electrode F2 of right frontal region. **b** Average ERP waveforms of N400 for positive (black line), negative (red line) and neutral word (blue line) in pleasant, unpleasant and no odor context recorded at electrode C1 of left central region. **c** Average ERP waveforms of N400 for positive (black line), negative (red line) and neutral word (blue line) in pleasant and unpleasant odor context recorded at electrode C2 of right central region. (Color figure online)

A 3 × 3 × 4 repeated measures ANOVA for N400 component amplitude showed a significant main effect for emotional words (F (2, 92) = 10.925, *p* < 0.001, η_p_^2^ = 0.192), yet not for odor context [F (2, 46) = 0.408, *p* = 0.668, η_p_^2^ = 0.017]. Further analyses indicated that the neutral words (−0.59 ± 0.26 μv) elicited a larger N400 than the positive (0.06 ± 0.27 μv, *p* < 0.001) and negative (−0.12 ± 0.23 μv, *p* = 0.003) words (Fig. 7). The main effects brain regions was also significant [F (3, 138) = 10.484, *P* < 0.001, η_p_^2^ = 0.186], with greater amplitude observed in the left frontal region (−0.61 ± 0.26 μv) than in the right frontal region (−0.09 ± 0.27 μv, *p* < 0.001) or right central region (0.17 ± 0.27 μv, *p* = 0.001). Meanwhile, the amplitude of the left central region (−0.325 ± 0.23 μv) was larger than the amplitude of the right central region (0.17 ± 0.27 μv, *p* = 0.001). There was a significant interaction between emotional words and odor context [F (4, 92) = 2.582, *p* = 0.042, η_p_^2^ = 0.101]. A simple effect analysis demonstrated that the neutral words (−0.73 ± 0.45 μv) induced a larger N400 amplitude than the positive words (0.42 ± 0.46 μv) under a pleasant odor context (*p* < 0.001) (Fig. 8). The interaction of emotional words × odor context × region was also significant [F (12, 276) = 2.098, *p* = 0.017, η_p_^2^ = 0.084]. In the left frontal region (*p* = 0.003) and the right frontal region (*p* = 0.001), neutral words (left: −1.10 ± 0.49 μv; right: −0.51 ± 0.51μv) in a pleasant odor context induced a greater N400 amplitude than the positive words (left: −0.021 ± 0.52 μv; right: 0.621 ± 0.51 μv). In the left central region, neutral words (−0.37 ± 0.45 μv) in the no odor context induced a larger N400 amplitude than the positive words (0.29 ± 0.47 μv, *p* = 0.030). In the pleasant odor context, positive words (0.32 ± 0.44 μv) induced a smaller N400 amplitude than the negative (−0.42 ± 0.39 μv) and neutral words (−1.06 ± 0.42 μv, *p* < 0.05), and negative words (−0.42 ± 0.39 μv) also induced a smaller EPN amplitude than the neutral words (−1.06 ± 0.42 μv, *p* = 0.015). Under the unpleasant odor context, the amplitude of N400 induced by the negative words (−0.23 ± 0.39 μv) was smaller than that of the neutral words (−0.88 ± 0.42 μv, *p* = 0.015). In the right central region, the N400 amplitude induced by the positive words (0.77 ± 0.47 μv) in the pleasant odor context was smaller than that induced by the neutral words (−0.26 ± 0.49 μv, *p* = 0.001). Under the unpleasant odor context, the amplitude of N400 induced by the negative words (0.28 ± 0.46 μv) was smaller than that for the neutral words (−0.60 ± 0.49 μv, *p* = 0.004) (Fig. 9). However, the other main effects and interactions were not significant (all ps > 0.05).

#### LPP (450–650 ms)

A 3 × 3 × 4 repeated measures ANOVA for LLP component amplitude in both brain hemispheres revealed a significant main effect for emotional words [F(2, 92) = 8.482, *p* < 0.001, η_p_^2^ = 0.156] and for brain region [F(3, 138) = 8.493, *p* < 0.001, η_p_^2^ = 0.156], yet not for odor context [F(2, 46) = 0.790, *p* = 0.460, η_p_^2^ = 0.033]. Further analyses indicated that the negative words (2.15 ± 0.33 μv) elicited a larger LPP than the neutral (1.65 ± 0.31 μv, *p* = 0.003) or positive words (1.72 ± 0.31 μv, *p* = 0.001) (Fig. 7). In addition, the amplitude of the right frontal region (1.97 ± 0.33 μv, *p* = 0.002) and the left central region (1.86 ± 0.31 μv, *p* = 0.014) were both larger than the left frontal region (1.33 ± 0.38 μv). Meanwhile, no significant interactions between factors were observed (ps > 0.05).

## Discussion

In this study, ERP technology was combined with emotional word recognition tasks to investigate the neural mechanism(s) by which different olfactory contexts might affect the processing of emotional words. The results obtained suggested that emotional word processing had strong automaticity, and an odor context with the same valence as emotional words could better regulate cognitive processing of emotional words. Furthermore, this regulatory effect appeared to start after 160 ms of early emotional word processing, and lasted up to 450 ms into late emotional word processing.

### Odor environment reduces the reaction time of emotional words at the behavioral level

We initially observed that negative words were more accurately recognized than positive words by our cohort, which indicated a "negative bias" in word recognition. Previous studies had also found that individuals tended to pay greater attention to negative words (Jia and Zhiru 2007; Kissler et al. 2009). One possible reason was that words with a negative valence were more threatening, which might involuntarily allocate more attention resources and lead to higher accuracy. We also observed that the recognition time of positive versus negative words was shorter than that of neutral words. This result demonstrated the “emotional effect” of word processing. For example, emotional words (such as “love”) were processed more quickly and accurately than neutral words (such as “train”) (Eviatar et al. 1990; Kissler et al. 2006). Secondly, our reaction time results demonstrated that the odor of an environment had an impact on the speed of word recognition (the emotional valence of words was not distinguished). Compared with a background with no odor, the presence of pleasant or unpleasant odors shortened the reaction time of recognizing words. Thus, special odors could provide an advantage in the recognition of words. Due to the high adaptability of a human’s sense of smell (Dalton 2000), we scored pleasantness, intensity, and arousal of each subject’s environment before and after each experimental task in order to exclude the possibility that a subject’s odor perception could decrease with time. We found no difference between the scores obtained before and after the tasks performed in the three odor environments. Therefore, our results indicated that the main effect of the order was not significant, which proved that our control over the concentration of the three odor environments was stable. This result was consistent with work by Seubert et al. (2010), which demonstrated that any odor might accelerate the speed of a behavioral response in facial emotion recognition tasks (Seubert et al. 2010). Another possible reason for the ability of a pleasant or unpleasant odor context to speed up a person's reaction to words was that pleasant odors and unpleasant odors were equally more arousing than neutral odors. Thus, if an odor environment was more stimulating, then individuals exhibited greater focus on a task and they reacted faster. Damasio (1996) also proposed that the orbitofrontal cortex (OFC) reactivated the emotional value of any type of object/context through body regulation, or via further coding in simulation of the cortical sensory region (Damasio 1996). Consequently, an odor environment might arouse emotional and sensorimotor regions of the brain, thereby affecting an individual’s response to words, including shortening the behavioral response time to words.

However, the accuracy and reaction time results all showed that the odor context did not have a matching effect on the accuracy and speed of different emotional word recognition, which was different from the previous behavioral results that the recognition speed of fearful faces was faster than happy faces under the unpleasant odor context (Li et al. 2020). The reason for the different results might be that words and pictures were different forms of expressing emotions, resulting in different emotional intensity. Pictures might be more visually prominent and/or processed faster, and word might require more auxiliary resources (Wood et al. 2016). Another possible reason was that although the setting of the odor concentration kept the subjects’ sensitivity relatively stable during the experiment, it was not enough to make the word with slow processing speed get fast valence consistency matching and fast button response. Therefore, there was no significant interaction result in behavioral indicators.

### Phases of the processing of emotional words

Our ERP results showed that the main effect of emotional words was significant in the middle processing period (200–300 ms). In addition, negative words induced a greater EPN amplitude than both positive and neutral words, indicating a "negative bias" in word processing. Many studies had suggested that EPN reflected an individual's classification of emotions, and stimuli with emotional significance could obtain greater selective attention. Therefore, compared with neutral stimuli, emotional stimuli induced EPN with a larger amplitude (Herbert et al. 2008; Kanske and Kotz 2007; Kissler et al. 2007; Schacht and Sommer 2009b). It had also been proposed that the amplitude of EPN was regulated by the degree of emotional arousal. Correspondingly, threatening and repulsive messages had been found to be more effective at attracting attention and eliciting greater electrophysiological activation (Lang and Bradley 2010; Ohman et al. 2001; Vuilleumier 2005).

Positive and negative words also induced a smaller N400 amplitude than neutral words in the present study. N400 reflected the integration process of lexical access (Lau et al. 2008) and lexical semantics (Kutas and Federmeier 2000). A smaller N400 amplitude indicated that semantic processing of emotional words was easier (Cao and Wang 2018). This result verified the research results of Kanske and Kotz (2007) which described that emotional words were generally processed faster in a semantic analysis than neutral words, and they induced N400 with a smaller amplitude during the late stage of word processing (Kanske and Kotz 2007). Thus, emotional words appeared to have priority in a semantic analysis, or emotional words were more easily accessible than neutral words (Kanske and Kotz 2007). The results of the present study indicated that emotional words had greater social significance to individuals, and their semantic meanings could be acquired more quickly than neutral words. Emotional nature provided obvious processing advantages.

During the late processing stage, negative words induced greater LPP amplitude than positive and neutral words, again reflecting the "negative bias" of word processing. This result indicated that at the late stage of emotional word processing, compared with positive and neutral words, individuals continued to pay more attention to negative emotional words. Moreover, negative emotional words induced a deeper emotional meaning assessment process (Fischler and Bradley 2006; Herbert et al. 2006). Regarding the negative bias, it was possible that a negative stimulus carried greater information value than a positive stimulus (Peeters and Czapinski 1990). Thus, even if there was no explicit task to promote an individual’s attention to its input, negative incentives would automatically capture more attention and greater cognitive resources than neutral or positive stimuli. As a result, a deeper, more sophisticated coding of the processing was achieved.

### Phases of the effect of the olfactory context on the processing of emotional words

#### Global effects of the odor context on the processing of emotional word

It had been observed that pleasant and unpleasant odors induced greater P2 amplitude in the early stages of word processing than neutral odors, regardless of the emotional valence of the words. This effect was consistent with that observed in previous studies that at least one stage of smell-visual integration involved nonspecific effects of odors on facial expression processing (Forscher and Li 2012; Leleu et al. 2015; Rubin et al. 2012). There were two possible explanations for this result. First, it was possible that an odor environment, whether positive or negative, aroused people's motivation to approach or avoid, respectively. Thus, in the early stage of emotional word recognition, our arousal and neural excitability were improved under the emotional odor context. As a result, individuals with excited nerves paid more attention to other information in their environment (emotional words), and this ultimately affected their speed of word recognition. A second possibility was that the OFC might contribute to reactivation of the emotional value of any type of object/context through body regulation, or through further coding with stimulation of the cortical sensory region (Damasio 1996). The somatosensory cortex also played an important role in facial expression recognition (Adolphs 2002; Pourtois et al. 2004). Therefore, an odor environment might activate both emotional and sensorimotor regions, thereby altering an individual’s response to subsequent words. Further research needed to precisely determine whether these brain regions were involved in the visual processing of emotional words that was influenced by odor within the time frame identified in the present study.

#### Specific effect of odor context on the processing of emotional word

In the present study, interactions between odor context and emotional words were identified in the early P2 component and the late N400 component of word processing. In the presence of an unpleasant odor, recognition of negative words with the same odor valence was found to induce a greater P2 amplitude than that by positive words. The P2 effect was associated with automatic processing of emotions, regardless of the depth of word processing (Begleiter and Platz 1969). These results suggested that in the presence of an unpleasant odor, negative words which matched the odor valence were rapidly processed at an early stage, greater P2 amplitude was provoked, which reflected a consistent promotion effect. Thus, with the unpleasant odor context inducing specific regulation of emotional word processing during its early stages, a promotion effect of valence consistency was observed.

In the late stage of emotional word processing, neutral words were found to induce a larger N400 amplitude than positive words in the absence of an odor context. This result was consistent with other results by previous studies (Kanske and Kotz 2007), that was, in previous studies about the word processing (in which the default was a no odor environment), the semantic analysis process of emotional words was usually faster than that of neutral words at the late stage of word processing. In addition, emotional words induced a smaller N400 amplitude than neutral words. N400 reflected the integration process of lexical access (Lau et al. 2008) and lexical semantics (Kutas and Federmeier 2000). A smaller N400 amplitude indicated that semantic processing of emotional words was easier (Cao and Wang 2018). Therefore, the present results indicated that emotional words had priority in a semantic analysis, or that emotional words were more easily understood than neutral words (Kanske and Kotz 2007). Secondly, the present results showed that in the left and right frontal regions of the brain, exposure to positive words in a pleasant odor context resulted in a smaller N400 amplitude compared with neutral words. In the left central region, the N400 amplitude caused by positive words was smaller than that caused by negative or neutral words in the pleasant odor context. Meanwhile, under the unpleasant odor context, the amplitude of N400 induced by negative words was smaller than that of neutral words. In the right central region, the N400 amplitude caused by positive words in the pleasant odor context was smaller than that caused by neutral words. Under the unpleasant odor context, the amplitude of N400 induced by negative words was smaller than that of neutral words. Taken together, the results of this study demonstrated that, especially in the context of pleasant or unpleasant odors, emotional words which had the same odor valence were recognized semantically more quickly, and it showed obvious processing advantages. Therefore, it was proved that pleasant and unpleasant odor contexts produced specific regulation on emotional words in the late processing stage, and the regulation mechanism was consistent with the promotion effect of valence consistency.

There were limitations associated with this study. For example, different emotional words had different parts of speech and were also affected by concreteness. Concrete words were responded to more quickly and accurately than abstract words (Groot and Annette 1989; Paivio and Allan 1991). We did not take concreteness into account when selecting the emotional words used. In future studies, the part of speech, arousal, and concreteness of emotional words should be considered as independent variables to study the influence of odor on emotional words and its processing mechanism. Subsequent studies could also address whether differences in the word recognition response which are caused by different odor environments are mediated by somatosensory areas. And it will be of interest to determine whether the somatosensory areas can communicate emotions between emotional regions of the brain such that different odors would induce distinct activation levels of the somatosensory areas, and thus respond differently to visual emotional objects.

## Conclusion

This study explores the processing mechanism of emotional words and the influence of odor context on visual processing of emotional words. Both our behavioral and EEG results demonstrate the emotional effects of words. More importantly, it was observed that the speed of emotional word recognition was affected by the external odor environment. Thus, subjects recognized emotional words faster in the context of pleasant or unpleasant odors. The ERP results further show two modes and corresponding time stages of emotional word processing that were regulated by odor context. The early P2 components of word processing exhibited that both pleasant odor and unpleasant odor contexts enhance neural excitability, and mediate undifferentiated effects in the processing of various emotional words. This may be related to the motivation of inducing tendency and avoidance. Meanwhile, in the early P2 stages of word processing, the consistency promotion effect of odor-emotional words was shown in the pleasant odor context. Finally, in the late N400 stage, the consistency promotion effect of odor-emotional words also was observed in both pleasant and unpleasant odor environments. Taken together, the complex mechanism of the effect of odor context on the processing of emotional words was mainly divided into the promoting effect of emotional odor contexts at the early stage of processing and the consistency promotion effect of odor-emotional word at the early and late stage. Use of brain imaging techniques and the unique role played by somatosensory areas are of particular interest for further studies.

## Data Availability

The datasets generated for this study are available on request to the corresponding author.
